# Early assessment of diffusion and possible expansion of SARS-CoV-2 Lineage 20I/501Y.V1 (B.1.1.7, variant of concern 202012/01) in France, January to March 2021

**DOI:** 10.2807/1560-7917.ES.2021.26.9.2100133

**Published:** 2021-03-04

**Authors:** Alexandre Gaymard, Paolo Bosetti, Adeline Feri, Gregory Destras, Vincent Enouf, Alessio Andronico, Sonia Burrel, Sylvie Behillil, Claire Sauvage, Antonin Bal, Florence Morfin, Sylvie Van Der Werf, Laurence Josset, ANRS MIE AC43 COVID-19, French viro COVID group, François Blanquart, Bruno Coignard, Simon Cauchemez, Bruno Lina, ANRS MIE AC43 COVID-19, ANRS MIE AC43 COVID-19, Elyanne Gault, Frédérique Moreau, Ségolène Brichler, Héloïse Delagrèverie, Diane Descamps, Charlotte Charpentier, Flore Rozenberg, Anne-Sophie L'Honneur, David Veyer, Laurent Bélec, Slim Fourati, Christophe Rodriguez, Jean-Michel Pawlotsky, Jacques Fourgeaud, Hanène Abid, Anne-Marie Roque-Afonso, Honorine Fenaux, Aude Jary, Stéphane Marot, Maud Salmona, Marie-Laure Chaix, Laurence Morand-Joubert, Aurélie Schnuriger, Stéphanie Marque Juillet, Pauline Bargain, Cécile Poggi, Lionel Chollet, Clémence Guillaume, Jérôme Guinard, Sophie Vallet, Léa Pilorgé, Evelyne Schvoerer, Cédric Hartard, Sandrine Castelain, Catherine François, Alexandra Ducancelle, Caroline Lefeuvre, Quentin Lepiller, Solène Marty-Quinternet, Pantxika Bellecave, Camille Tumiotto, Julia Dina, Meriadeg Le Gouil, Cécile Henquell, Audrey Mirand, Raymond Césaire, Alexis de Rougemont, Christelle Auvray, Sylvie Larrat, Benjamin Némoz, Claire Tinez, Aurélie Guigon, Sébastien Hantz, Sylvie Rogez, Georges Dos Santos, Pascale Perez, Christelle Jost, Brigitte Montes, Vincent Foulongne, Berthe-Marie Imbert, Céline Bressollette, Valérie Giordanengo, Géraldine Gonfrier, Magali Garcia, Nicolas Lévêque, Véronique Brodard, Hélène Moret, Vincent Thibault, Anne Maillard, Marie-Christine Jaffar-Bandjee, Marie Gueudin, Jean-Christophe Plantier, Bruno Pozzetto, Sylvie Pillet, Samira Fafi-Kremer, Morgane Solis, Jacques Izopet, Pauline Trémeaux, Karl Stefic, Lynda Handala, Geneviève Billaud, Emilie Frobert, Audrey Mérens, Christine Bigaillon, Marine Desroches, Cédric Thepenier, Frédéric Janvier, Marie-Pierre Otto, Bénédicte Roquebert, Stéphanie Haïm-Boukobza, French viro COVID group, French viro COVID group

**Affiliations:** 1 CNR des virus des infections respiratoires (dont la Grippe), Institut des Agents Infectieux, Hopital de la Croix Rousse, HCL, Lyon, France; 2 Centre International de recherche en infectiologie (CIRI), Virpath Team, Inserm U1111, CNRS UMR5308, École Normale Supérieure de Lyon, UCBL, Université de Lyon, Lyon, France; 3 These authors contributed equally; 4 Mathematical Modelling of Infectious Diseases Unit, Institut Pasteur, UMR 2000, CNRS, Paris, France; 5 Santé Publique France, Direction des maladies infectieuses, Saint-Maurice, France; 6 CNR des virus des infections respiratoires (dont la Grippe), Molecular Genetics of RNA Viruses, CNRS UMR 3569, Institut Pasteur, Université de Paris, Paris, France; 7 GHU Pitié-Salpêtrière APHP, 83, boulevard de l’hôpital & SU-INSERM UMR_S 1136 Team 3 THERAVIR IPLESP, Paris, France; 8 The members of the group are listed under Investigators; 9 Centre for Interdisciplinary Research in Biology (CIRB), Collège de France, CNRS, INSERM, PSL Research University, Paris, France; 10 Infection Antimicrobials Modelling Evolution, UMR 1137, INSERM, Université de Paris, Paris, France; 11 These senior authors contributed equally

**Keywords:** COVID, SARS-CoV-2, 20I/501Y.V1, France, surveillance, viral infections, mutation

## Abstract

The emergence of SARS-CoV-2 variant 20I/501Y.V1 (VOC-202012/1 or GR/501Y.V1) is concerning given its increased transmissibility. We reanalysed 11,916 PCR-positive tests (41% of all positive tests) performed on 7–8 January 2021 in France. The prevalence of 20I/501Y.V1 was 3.3% among positive tests nationwide and 6.9% in the Paris region. Analysing the recent rise in the prevalence of 20I/501Y.V1, we estimate that, in the French context, 20I/501Y.V1 is 52–69% more transmissible than the previously circulating lineages, depending on modelling assumptions.

The emergence of a variant of severe acute respiratory syndrome coronavirus 2 (SARS-CoV-2), called VOC-202012/1 (lineage B.1.1.7, 20I/501Y.V1 or GR/501Y.V1) and first observed in the United Kingdom (UK), is a major concern for the management of the corona virus disease (COVID-19) pandemic [1]. It is essential to assess the current and future circulation of this variant in Europe.

## A nationwide survey of 501Y.V1 in France

The SARS-CoV-2 variant 20I/501Y.V1 (501Y.V1) contains a deletion at position 69–70 of the spike (S) protein in the target region of the ThermoFisher TaqPath PCR probe targeting the S gene that leads to a loss of amplification [2]. In December 2020, the first variants with S-gene target failure (SGTF) were detected in France through the use of the TaqPath RT-PCR (Scientific TaqPath COVID-19 Combo Kit, Thermo Fisher, Waltham, United States (US)). Since some viruses of the European lineage circulating in France can also harbour the S 69–70 deletion (20A, 20A(EU2), 20E(EU2)), the circulation of the 501Y.V1 variant needed to be assessed by sequencing of the SGTF viruses. The first case of infection with 501Y.V1 was detected on 13 December 2020. By the end of December, 38% (n = 87) of the SGTF viruses detected by the TaqPath RT-PCR had been confirmed as 501Y.V1 by sequencing [3], with a slowly increasing trend. However, the surveillance was not able to provide a robust picture of the circulation of the variant in France.

To assess the level of circulation of 501Y.V1, a nationwide survey (called Flash#1) was implemented on 7 and 8 January. Briefly, all private and public diagnostic laboratories in Metropolitan France were asked to participate to the study on a voluntary basis by providing to the National Reference Centre the number of SARS-CoV-2 PCR tests carried out during these 2 days and the number of PCR-positive tests. In addition, the laboratories were asked to test all their SARS-CoV-2 PCR-positive specimens with the TaqPath Kit. Subsequently, all SGTF specimens were sequenced for confirmation of lineage.

During the 2-day survey, we also collected the total number of SARS-CoV-2 diagnostic tests performed by RT-PCR and the number of positive tests in France to assess the representativeness of the survey.

## Level of circulation of 501Y.V1 across France

Overall, 135 laboratories located in all regions of France contributed to the Flash#1 survey (Table 1). A total of 183,363 RT-PCR tests were included in the survey, with 11,916 positive. This represented 36% of all SARS-CoV-2 PCRs performed in France during these 2 days, and 41% of the PCR-positive tests reported in France during this period. Among the 11,916 positive tests, 552 (4.6%) had the SGTF profile. Of those, 424 (76.8%) were successfully sequenced either by Sanger sequencing (S gene) or whole genome sequencing (WGS; Illumina, San Diego, US). The sequencing detected 298 cases with 501Y.V1 viruses among the 424 (70.3%). As a consequence, we estimate that 70.3% of the 552 SGTF viruses were 501Y.V1 viruses, representing 3.3% of all SARS-CoV-2 detections (Table 2).

**Table 1:** National results of the Flash#1 survey, SARS-CoV-2 diagnostic testing, France, 7–8 January 2021 (n = 183,363 samples)

Number of laboratories	135
Total number of samples	183,363
Number of RT-PCR positive samples	11,916
Number of samples with S-gene target failure (SGTF)	552
Number of samples sent for sequencing	482
Number of samples successfully sequenced	424
Number of 501Y.V1 sequences	298

SARS-CoV-2: severe acute respiratory syndrome coronavirus 2.

**Table 2:** Regional results of the Flash#1 survey, SARS-CoV-2 diagnostic testing, France, 7–8 January 2021 (n = 11,916 samples)

Region	RT-PCR positive (n)	RT-PCR with SGTF (n)	Samples sent for sequencing (n)	Samples successfully sequenced (n)	501Y.V1 sequences (n)	Proportion of confirmed 501Y.V1 among all the successfully sequenced samples (%)	Estimated proportion of 501Y.V1 cases (%) ^a^
Auvergne-Rhône-Alpes	2,405	68	60	46	26	56.5%	1.6%
Bourgogne-Franche Comté	585	39	38	37	1	2.7%	0.2%
Brittany	307	18	7	7	1	14.3%	0.8%
Centre-Val de Loire	523	23	23	20	16	80.0%	3.5%
Grand Est	805	40	30	18	4	22.2%	1.1%
Hauts de France	482	16	11	9	7	77.8%	2.6%
Ile-de-France	2,149	158	145	132	124	93.9%	6.9%
Nouvelle Aquitaine	512	13	3	3	2	66.7%	1.7%
Normandy	428	9	9	9	5	55.6%	1.2%
Occitanie	339	10	8	4	4	100.0%	2.9%
Provence-Alpes-Côte d'Azur	1,881	105	96	88	75	85.2%	4.8%
Pays de la Loire	513	19	18	17	6	35.3%	1.3%
France (not attributable) ^b^	987	34	34	34	27	79.4%	2.7%
Total Metropolitan France (without Corsica)	11,916	552	482	424	298	70.3%	3.3%

SARS-CoV-2: severe acute respiratory syndrome coronavirus 2; SGTF: S-gene target failure.

^a^ This estimate is calculated by applying the proportion of confirmed 501Y.V1 among all the successfully sequenced samples to the fraction of RT-PCR with SGTF over all the RT-PCR positives.

^b^ Results from several laboratories processing samples from metropolitan France.

**Figure 1:**
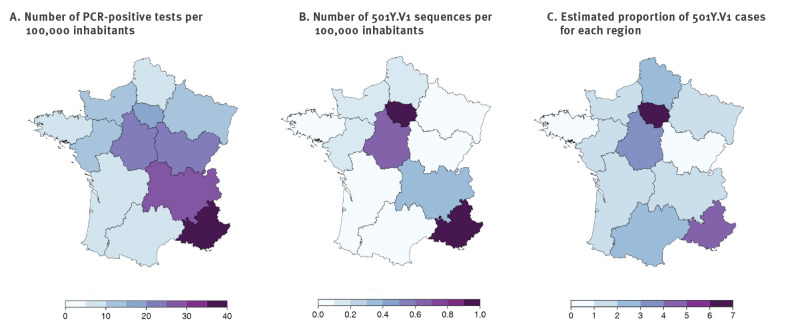
Distribution of 501Y.V1 cases by location of sampling laboratories, Flash#1 survey, France, 7–8 January 2021

Regional disparities were observed. The prevalence of 501Y.V1 among cases ranged from 0.2% in the Bourgogne-Franche Comté region to 6.9% in Ile-de-France (Table 2 and Figure 1). In particular, about two thirds of 501Y.V1 were observed in Ile-de-France and Provence-Alpes-Côte d'Azur, the two regions which had the largest proportions of 501Y.V1 among samples (6.9% and 4.8%, respectively).

## Estimates of increased transmissibility of 501Y.V1 in France

**Figure 2:**
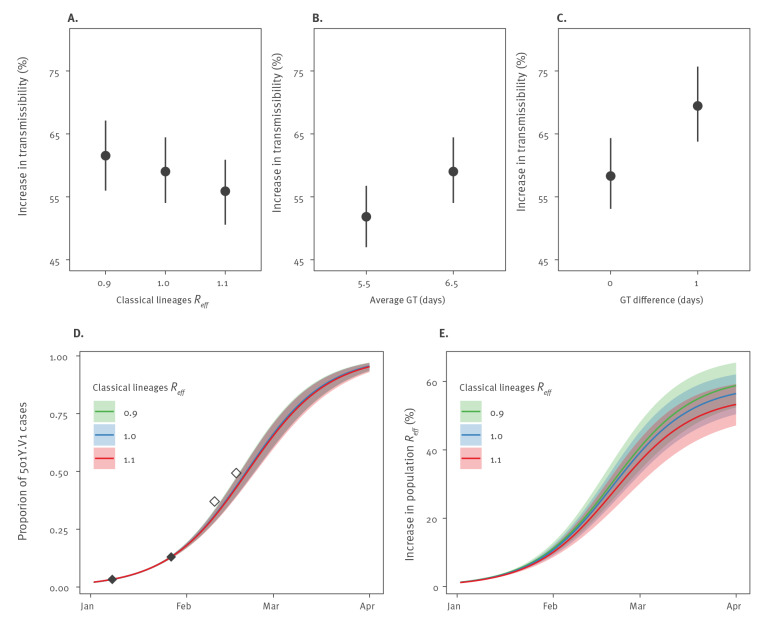
Estimated increase in transmissibility of the 501Y, Flash surveys, France, January 2021

GT: generation time; *R_eff_*: effective reproduction number; SARS-CoV-2: severe acute respiratory syndrome coronavirus 2.

A second survey (Flash#2) [4] was performed on 27 January 2021 and found a prevalence of 501Y.V1 of 13.0% (1,335 of 10,261 tests PCR-positive for SARS-CoV-2) on that date (Supplement). We analysed the growth in the prevalence of 501Y.V1 between Flash#1 and Flash#2 to estimate the increased transmissibility of 501Y.V1 relative to the classical European lineage viruses. In our baseline scenario, we assume that the effective reproduction number (*R_eff_*) of the classical lineages was 1.0 on average between the surveys [5] and that all viruses had a gamma-distributed generation time with a mean of 6.5 days and a coefficient of variation of 0.62 [1]. We estimated that the 501Y.V1 variant was 59% (95% credible interval (CrI): 54–65%) more transmissible than the classical lineages, consistent with estimates from the UK [1] (Figure 2A). In sensitivity analyses, we showed that the estimated competitive advantage of 501Y.V1 would be little affected by changes in our assumptions about the *R_eff_* of the classical lineages during the study period (Figure 2A). A lower generation time with a mean of 5.5 days and a coefficient of variation of 0.33 for both viruses would reduce the competitive advantage to 52% (95% CrI: 47–57%) (Figure 2B). Estimates of the competitive advantage would increase to 69% (95% CrI: 64–76%) if the generation time of 501Y.V1 was 1 day longer than that of the classical lineages [6] (Figure 2C).

A–C. Increased transmissibility of 501Y.V1 variant relative to the classical European lineages, under different assumptions for the GT distribution and the *R_eff_* of the classical European lineages.

A. GT distribution with a mean of 6.5 days and a coefficient of variation of 0.62 for both viruses (baseline) for *R_eff_* ranging from 0.9 to 1.1.

B. Comparing the baseline estimates to those obtained using a GT distribution with a mean of 5.5 days and coefficient of variation of 0.33 for both viruses and for *R_eff_* = 1.0.

C. Increasing the mean GT of the variant from 6.5 (GT difference = 0) to 7.5 (GT difference = 1).

D. Temporal trends for the proportion of 501Y.V1 among SARS-CoV-2 cases.

E. Temporal trends for the expected increase in the effective reproduction number of a person infected with SARS-CoV-2 (averaged across the different variants) in France relative to a scenario where 501Y.V1 would not be circulating in France.

The trends are shown for three values of *R_eff_* (0.9 in green, 1.0 in blue, and 1.1 in red). In panels A, B and C, dots represent posterior means while vertical bars represent 95% credible intervals. In panels D and E, solid lines represent posterior means while ribbons represent 95% credible intervals. In panel D, filled diamonds represent data from Flash#1 and Flash#2 used for model calibration; empty diamonds are external validation data (not used for model calibration).

We used these estimates to assess future trends of the proportion of 501Y.V1 infections in France, considering different scenarios for the *R_eff_* of the previously circulating lineages, ranging from 0.9 to 1.1 for the coming months. For *R_eff_* = 1.0, we estimated that the proportion of 501Y.V1 cases would reach 66% (95% CrI: 61–71%) and 96 (95% CrI: 94–97%) by 1 March and 1 April 2021, respectively (Figure 2D). The predicted trajectory closely matched two recent estimates of the prevalence of 501Y.V1 that were not used for inference (Figure 2D) [7,8] (Supplement).

As the prevalence of 501Y.V1 increases, we expect that the population-level *R_eff_* (i.e. the one averaged across the different variants) will be respectively 39% (95% CrI: 33–45%) and 56% (95% CrI: 50–62%) higher on 1 March and 1 April 2021 than what would be expected if only the classical lineages were circulating (Figure 2E). These results were little affected when we changed the values for the *R_eff_* of the previously circulating lineages (Figure 2 D and E).

## Conclusion

This first round of investigation has emphasised the need for strengthening the SARS-CoV-2 genomic surveillance through rapid and accurate monitoring of current and future variants. As a consequence, repeated flash surveys are now scheduled, and a national SARS-CoV-2 genomic surveillance scheme coordinated by Santé publique France, the national research agency for AIDS and viral hepatitis/emerging infectious diseases (Agence nationale de recherches sur le sida et les hépatites virales/Maladies infectieuses émergentes (ANRS/MIE)) and the National Reference Laboratory for respiratory viruses (including influenza) has been implemented, based on the reinforcement of four sequencing platforms to increase national sequencing capacities and accelerate sequence determination. In addition, the French health authorities promote the implementation of PCR-specific tools (detection of the 501Y and 484K single nucleotide polymorphisms) to enhance the screening capacity of laboratories. Further, randomly selected specimens will be analysed by the sequencing platforms. This strategy will address two complementary objectives, improved monitoring and real-time measurement of the impact of existing variants and rapid detection of newly emerging variants. In parallel, mathematical models anticipate how the rise of 501Y.V1 and other variants may affect the course of the pandemic and the impact of control measures [9,10]. It will also be important to determine how spatial heterogeneities in the spread of variants may affect control strategies.

## Supplementary Data

457000 Supplement
