# Correction for Euro Surveill. 2020;26(9)

**DOI:** 10.2807/1560-7917.ES.2021.26.10.210311c

**Published:** 2021-03-11

**Authors:** Eurosurveillance editorial team

**Affiliations:** 1 European Centre for Disease Prevention and Control (ECDC), Stockholm, Sweden

In the article ‘Early assessment of diffusion and possible expansion of SARS-CoV-2 Lineage 20I/501Y.V1 (B.1.1.7, variant of concern 202012/01) in France, January to March 2021’ by Gaymard et al., published on 4 March 2021, a recognition of the laboratories belonging to the ColVHB network was added to the Acknowledgements section. This change was made on request of the authors on 5 March 2021.

