# Supplementary material for: Early assessment of diffusion and possible expansion of SARS-CoV-2 Lineage 20I/501Y.V1 (B.1.1.7, variant of concern 202012/01) in France, January to March 2021
Source: Euro Surveill. 2021 Mar 4;26(9):2100133. doi: 10.2807/1560-7917.ES.2021.26.9.2100133 (PMC7934223; doi:10.2807/1560-7917.ES.2021.26.9.2100133)
Supplement: Supplement [file 21-00133_GAYMARD_Supplement.pdf]

Supplement - Early assessment of diffusion and possible expansion of SARS-CoV-2 Lineage 20I/501Y.V1 (B.1.1.7, Variant of Concern 202012/01) in France, January to March 2021

Gaymard et al, Eurosurveillance

This supplementary material is hosted by Eurosurveillance as supporting information alongside the article "Early assessment of diffusion and possible expansion of SARS-CoV-2 Lineage 20I/501Y.V1 (B.1.1.7, Variant of Concern 202012/01) in France, January to March 2021", on behalf of the authors, who remain responsible for the accuracy and appropriateness of the content. The same standards for ethics, copyright, attributions and permissions as for the article apply. Supplements are not edited by Eurosurveillance and the journal is not responsible for the maintenance of any links or email addresses provided therein.

Statistical Model

In order to estimate the increase in transmissibility of the 501Y.V1 variant with respect to the historical lineages, we use Markov Chain Monte Carlo sampling with a likelihood given by:

$$L = \prod_t \quad Binom(D(t);\ N(t), p(t))$$

where $D(t)$ is the number of sequences where the 501Y.V1 variant was detected, $N(t)$ is the total number of positive sequences, $p(t)$ is the expected proportion of 501Y.V1, and t the time point - corresponding to the two surveys discussed in the main text.

We assume a simple exponential growth model according to which $p(t)$ can be computed as follows:

$$p(t) \ = 1/(1 + \frac{1 - p_0}{p_0} e^{(r_H - r_V)t})$$

where r_H and r_V are the growth rate of the historical lineages and 501Y.V1 variant, and p_0 is the proportion of 501Y.V1 at time $t = 0$ (which corresponds to January 1st 2021 in our model).

The growth rate can be obtained from the reproduction number by using the relation:

$$R \ = \ (1 + r/b)^a$$

where $a = 1/cv^2$ and $b = a/\mu$, with μ and cv the generation time distribution mean and coefficient of variation, respectively (Fraser et al., 2009). The reproduction number of the variant is given by:

$$R_v = (1 \ + \ \alpha) \ R_H$$

where R_H is the reproduction number of the historical lineages and α is the increase of transmissibility of the variant.

If R_H is fixed, then only two parameters need to be estimated: p_0 and α. We use non-informative, flat, priors for both of them.

Recent data on B.1.1.7 in France

The Flash#2 study was done on 27th January 2021 (Weekly epidemiological newsletter, SPF, February 11 2021). In short, 234 laboratories participated, including 120 335 RT-PCR tests of which 10 261 (8.5%) were positive. Ninety laboratories used the same protocol as in Flash#1: of 3 561 positive RT-PCRs, 465 (13.3%) had the SGTF profile. Of those, 312 were sequenced and 261 (83.6%) were confirmed 501Y.V1 infections by sequencing. If we apply this proportion to all S drop-out profiles, we estimate that 389 cases were infected by 501Y.V1 in this first group of laboratories. In addition, 144 laboratories used a screening RT-PCR specific to distinguish 501Y.V1 from 501Y.V2 and 501Y.V3 leading to the identification of 946 501Y.V1 infections. The positive predictive value of this screening strategy was 100%, as assessed by subsequent sequencing of the 501Y.V1 RT-PCR positive cases. Overall, we estimate 1335=389+946 cases were infected by 501Y.V1 out of 10 261 cases, corresponding to a prevalence of 13.0%.

The same screening RT-PCR specific for variants of concern V1, V2 and V3 is now used routinely, leading to the identification of 26 063 (37%) 501Y.V1 infections out of 70 498 tests from 9 to 15 February (Weekly epidemiological newsletter, SPF, February 18 2021) and 40 444 501Y.V1 infections (49%) out of 82 096 from 15 to 21 February (Weekly epidemiological newsletter, SPF, February 25 2021). The proportion of 501Y.V2 and 501Y.V3 variants is lower and represents 5% from 9 to 15 February and 5.6% from 15 to 21 February.

References

Fraser, Christophe, Christl A. Donnelly, Simon Cauchemez, William P. Hanage, Maria D. Van Kerkhove, T. Déirdre Hollingsworth, Jamie Griffin et al. "Pandemic potential of a strain of influenza A (H1N1): early findings." ***science*** **324, no. 5934 (2009): 1557-1561.**

Santé Publique France. COVID-19 Point épidémiologique hebdomadaire du 11 février 2021. [Weekly epidemiological newsletter, February 11 2021]. Paris: Santé Publique France; 2021. French. Available from:https://www.santepubliquefrance.fr/maladies-et-traumatismes/maladies-et-infections-respiratoires/infection-a-coronavirus/documents/bulletin-national/covid-19-point-epidemiologique-du-11-fevrier-2021

Santé Publique France. COVID-19 Point épidémiologique hebdomadaire du 18 février 2021. [Weekly epidemiological newsletter, February 18 2021]. Paris: Santé Publique France; 2021. French. Available from:https://www.santepubliquefrance.fr/maladies-et-traumatismes/maladies-et-infections-respiratoires/infection-a-coronavirus/documents/bulletin-national/covid-19-point-epidemiologique-du-18-fevrier-2021

Santé Publique France. COVID-19 Point épidémiologique hebdomadaire du 25 février 2021. [Weekly epidemiological newsletter, February 25 2021. Paris: Santé Publique

France; 2021. French. Available from:https://www.santepubliquefrance.fr/maladies-et-traumatismes/maladies-et-infections-respiratoires/infection-a-coronavirus/documents/bulletin-national/covid-19-point-epidemiologique-du-25-fevrier-2021
